# Age, exposure and immunity

**DOI:** 10.7554/eLife.40150

**Published:** 2018-08-21

**Authors:** Michael White, James Watson

**Affiliations:** 1 Department of Parasites and Insect VectorsInstitut PasteurParisFrance; 2 Mahidol Oxford Tropical Medicine Research Unit, Faculty of Tropical MedicineMahidol UniversityBangkokThailand; 3 Centre for Tropical Medicine and Global Health, Nuffield Department of MedicineUniversity of OxfordOxfordUnited Kingdom

**Keywords:** immunity, malaria, anti-parasite immunity, anti-disease immunity, <i>P. falciparum</i>

## Abstract

The acquisition of immunity to malaria by an individual depends on their age and the number of infectious mosquito bites they have received.

**Related research article** Rodriguez-Barraquer I, Arinaitwe E, Jagannathan P, Kamya MR, Rosenthal PJ, Rek J, Dorsey G, Nankabirwa J, Staedke SG, Kilama M, Drakeley C, Ssewanyana I, Smith DL, Greenhouse B. 2018. Quantification of anti-parasite and anti-disease immunity to malaria as a function of age and exposure. *eLife* **7**:e35832. doi: 10.7554/eLife.35832.

In a league table of infectious diseases, malaria would rank above all others in how often it causes infections and the number of parasites in infected hosts. In areas that are particularly favourable for malaria transmission, a single person can be infected more than 1,000 times per year (Smith et al., 2005). Each infection can potentially lead to a fatal episode of severe malaria that sees up to a trillion parasites circulating in the host’s blood.

Although every new infection boosts immunity to further infections, this comes at a high price: in some areas malaria can kill up to 5% of children before their fifth birthday (Streatfield et al., 2014). Adults become immune more quickly than children, but are also more likely to die from severe malaria (Baird, 1998). It would be useful, therefore, to be able to quantify the rate at which an individual acquires immunity to malaria, and how this depends on their age and exposure. Now, in eLife, Isabel Rodriguez-Barraquer of the University of California, San Francisco and colleagues in the US, Uganda and the UK report how they have developed a new model that sheds light on these relationships (Rodriguez-Barraquer et al., 2018).

Rodriguez-Barraquer et al. looked at data from 773 children aged between six months and 10 years old in three Ugandan villages. Each child was monitored for up to three years in an attempt to understand the relationships between their age, how often they were infected, the number of parasites in their blood, and the number of parasites required to cause symptoms of malaria. A child was considered to have developed a case of clinical malaria if they had a body temperature above 38°C, and if the density of parasites in their blood was high enough to be seen under a microscope. Alongside clinical follow-up, there was an accompanying effort to catch mosquitoes in the children’s houses. This allowed exposure to be measured in terms of the number of infectious mosquito bites the children received per year.

The villages had varying levels of malaria, with children experiencing an average of 22 bites and two cases of clinical malaria every year (Kamya et al., 2015). There was also a lot of variation in the number of cases per child, with one child experiencing 30 incidents of malaria over the course of the study.

Rodriguez-Barraquer et al. analysed data on two aspects of immunity – anti-parasite immunity (which reduces the number of parasites in the blood) and anti-disease immunity (which is the ability to tolerate a given parasite density without developing fever; Figure 1). They found that older children had fewer parasites than younger children, and that frequently infected children had fewer parasites than the less frequently infected. They also found that older children were less likely to develop fever than young children, and that frequently infected children were less likely to develop fever than the less frequently infected. These linear relationships agree with our current understanding of malaria immunity based on decades of epidemiological studies (Doolan et al., 2009).

**Figure 1.**
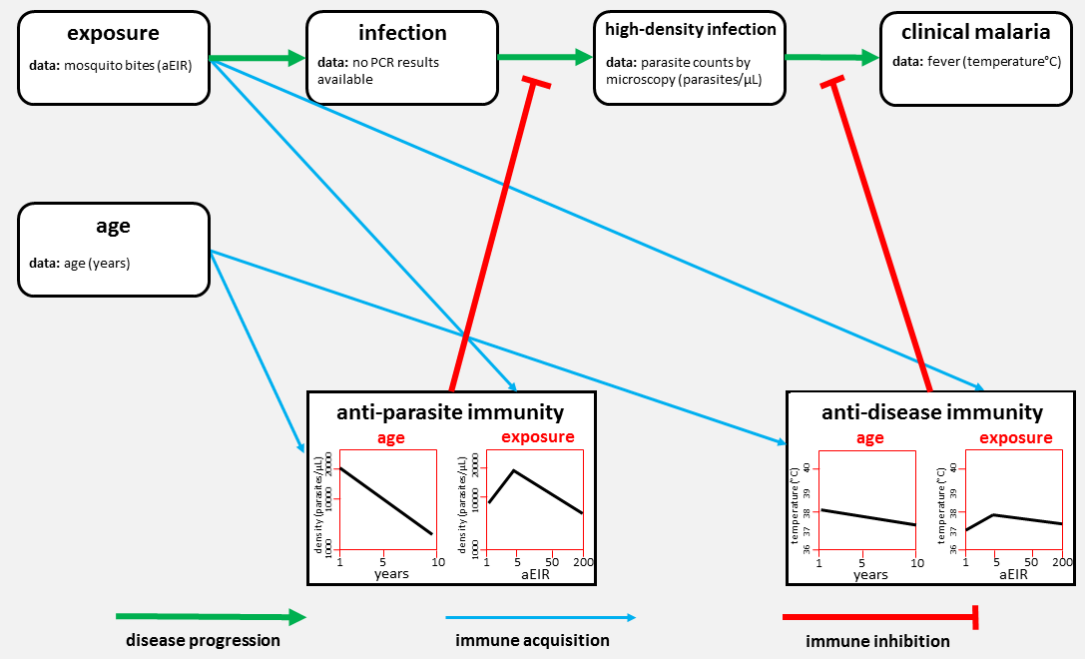
The network of factors used by Rodriguez-Barraquer et al. to model immunity to malaria. Malaria develops in stages (progression shown by green arrows). First, a mosquito bite can lead to a low-level infection (which can only be detected by techniques such as PCR). Rodriguez-Barraquer et al. focused on high-density infections, where there are enough parasites in the blood to be viewed using microscopy. These infections can lead to clinical malaria, which produces a fever. Two forms of immunity can inhibit the progression of the disease (red lines): anti-parasite immunity reduces the density of blood-stage infections, and anti-disease immunity reduces the temperature caused by a given parasite density. The plots show how each type of immunity varies with age (left) and exposure (right) in the model developed by Rodriguez-Barraquer et al. Exposure is measured in terms of the number of infectious mosquito bites per year, which is also known as the annual entomological inoculation rate (aEIR).

The researchers then investigated the nonlinear relationships between age, transmission and the acquisition of immunity. They found that children in the villages with the lowest rates of malaria transmission (who received an average of two infectious bites a year) developed immunity more efficiently than children in villages with moderate transmission rates (who received three times more bites). The average child coming to the health clinic in the low transmission village had both a lower parasite density and a lower body temperature than their counterpart from the moderate transmission village.

This is a counter-intuitive but statistically robust finding. If replicated elsewhere, it has important implications. Traditionally it has been assumed that as transmission rates decline, so does immunity, presenting an obstacle to malaria control (Rogier et al., 1999). However, the new analysis suggests a more complex pattern, which Rodriguez-Barraquer et al. suggest could be due to the lower genetic diversity found among parasites in low-transmission situations. This could mean that individuals may acquire immunity to infection more efficiently as malaria transmission is reduced, thus aiding control efforts. More work is needed to check if this association is causal and is free of selection bias. Selection bias could arise if the way children’s parents seek treatment varies for different transmission intensities.

The latest analysis also suggests future avenues of research. Asymptomatic infections, and those that do not reach sufficiently high density to be seen under a microscope, could be detected using PCR (polymerase chain reaction) techniques. Exposure could be measured more accurately by using genotyping to count the number of parasite clones circulating in the host (Mueller et al., 2012). Measurements of anti-malarial antibody responses can also be used as markers of both exposure to the disease and protection from it (França et al., 2017).

Levels of immunity are not just dependent on age and exposure, but also on the cumulative number of episodes of clinical malaria that an individual has experienced (Rodriguez-Barraquer et al., 2016). Other data sources may shed light on this problem. An ideal target would be to identify combinations of immune responses that allow immunity to be quantified independently of age and exposure to the malaria parasites.
